# Wood’s Medical and Surgical Monographs

**Published:** 1891-05

**Authors:** 


WOOD'S MEDICAL AND SURGICAL MONOGRAPHS. Vol. IX., No. 1, January, 1891; No. 2, February, 1891; and No. 3, March, 1891. William Wood & Co., 56 and 58 Lafayette place, New York. Monthly, $10.00 a year; single copies, $1.00.

The third year of this valuable series of monographs began with the January number, which contains: Advances in Bacteriology, by Robert Koch, M. D.; Formulary of New Remedies and New Medicinal Preparations, by H. Bocquillon-Limousin; and Anesthetics: A Discussion, by Dr. William Macewen and others.

The February number contains a chapter on the Clinical Use of Prisms and the Decentring of Lenses, by Ernest E. Maddox, M. B.; Electricity in the Treatment of Uterine Tumors, by Thomas Keith, M. D., LL. D., and Skene Keith, F. R. C. S.; and Ether-Drinking: Its Prevalence and Results, by Ernest Hart.

The March number contains: The Modern Diagnosis of Diseases of the Stomach, by Dr. J. M. Purser, Dublin; Unsoundness of Mind, in its Legal and Medical Considerations, by J. W. Hume Williams, London; Baldness and Greyness: Their Etiology, Pathology, Treatment, by Tom Robinson, M. D., London.

These three numbers, comprising Volume IX., fully maintain the previous high standard of these publications, and ought to find ready sale.

